# A Label-Free Microfluidic Biosensor for Activity Detection of Single Microalgae Cells Based on Chlorophyll Fluorescence

**DOI:** 10.3390/s131216075

**Published:** 2013-11-26

**Authors:** Junsheng Wang, Jinyang Sun, Yongxin Song, Yongyi Xu, Xinxiang Pan, Yeqing Sun, Dongqing Li

**Affiliations:** 1 College of Information and Science Technology, Dalian Maritime University, Dalian 116026, China; E-Mails: wangjsh@dlmu.edu.cn (J.W.); xuyongyi@dlmu.edu.cn (Y.X.); 2 College of Marine Engineering, Dalian Maritime University, Dalian 116026, China; E-Mails: golden_sun@126.com (J.S.); yongxin@dlmu.edu.cn (Y.S.); panxx@dlmu.edu.cn (X.P.);; 3 College of Environmental Science and Engineering, Dalian Maritime University, Dalian 116026, China; 4 Department of Mechanical & Mechatronics Engineering, University of Waterloo, Waterloo, ON N2L3G1, Canada

**Keywords:** label-free fluorescent sensor, microalgae cell activity, microfluidic chip, chlorophyll fluorescence

## Abstract

Detection of living microalgae cells is very important for ballast water treatment and analysis. Chlorophyll fluorescence is an indicator of photosynthetic activity and hence the living status of plant cells. In this paper, we developed a novel microfluidic biosensor system that can quickly and accurately detect the viability of single microalgae cells based on chlorophyll fluorescence. The system is composed of a laser diode as an excitation light source, a photodiode detector, a signal analysis circuit, and a microfluidic chip as a microalgae cell transportation platform. To demonstrate the utility of this system, six different living and dead algae samples (*Karenia mikimotoi* Hansen, *Chlorella vulgaris*, *Nitzschia closterium*, *Platymonas subcordiformis*, *Pyramidomonas delicatula* and *Dunaliella salina*) were tested. The developed biosensor can distinguish clearly between the living microalgae cells and the dead microalgae cells. The smallest microalgae cells that can be detected by using this biosensor are 3 μm ones. Even smaller microalgae cells could be detected by increasing the excitation light power. The developed microfluidic biosensor has great potential for *in situ* ballast water analysis.

## 1. Introduction

Ships' ballast water is one of the common sources of the oceanic biological invasions. Alien marine organisms have caused serious effects to the local biology, industry, agriculture, and human health around the World [1–5]. Consequently, discharged ship ballast waters must meet the requirements of the International Convention for the Control and Management of Ships' Ballast Water and Sediments (hereinafter referred to as the Ballast Water Convention) [6–9]. One of the most important criteria in this convention is that the concentration of the living organisms in ballast water cannot exceed a certain value (less than 10 per mL). Microalgae are typical living organisms in ships' ballast water, and they are the main targets for ballast water treatment and detection. The detection of algae cell activity (as an indication of cell's liveliness) and the determination of the concentration of live algae are necessary.

Major methods for detecting the activity of single microalgae and counting the algae in ballast water include optical microscopy, fluorescent microscopy, chlorophyll-a fluorometers and flow cytometry. In the optical microscope method, algae cell activity is judged by human eyes and it is usually laborious and inaccurate owing to the fact many living algae do not move. In the fluorescent microscope method, the algae must be stained by fluorescent dyes, a process that is time-consuming and laborious, and the efficiency of dye tagging often depends on the operator's skills. Chlorophyll-a fluorometers can accurately determine the chlorophyll content in cells, but they can only detect the chlorophyll fluorescence intensity of stationary cells and it cannot count the cells. The most popular instrument for detecting microalgae's physiological state is the flow cytometer which can count algae quantities automatically, however, dye staining is required [10,11]. Not all algae can be stained by one or even several dyes. In addition, commercial flow cytometers are relatively expensive and bulky so the technique is not suitable for use on ships.

Microfluidic chips or Labs-on-a-Chip (LOCs) are a promising technique for fast *in situ* detection of small amounts of samples. They are applied widely in chemistry, biology, medicine and the environment [12–23]. In recent years, impedance methods based on the Coulter principle have been used in LOC for detecting all kinds of microparticles [24–26]. In our previous studies [27], counting algae in ballast water has been accomplished by using a microfluidic resistance pulse sensor (RPS) technique, but this method cannot determine if a microalgal cell is alive or dead.

As the photosynthesis activity of a plant cell indicates the cell's liveliness, it can be used to determine if the algae cells are alive or dead. Chlorophyll is a key biomolecule allowing plants to absorb energy from light and very important in algal photosynthesis. In photosynthesis, some excess energy is emitted as light—chlorophyll fluorescence. Chlorophyll fluorescence intensity has been demonstrated to be proportional to chlorophyll contents in cells and can be used for evaluating the photosynthetic capacity in algae and plant cells [28–35]. Therefore, chlorophyll fluorescence intensity can be used to characterize microalgae cell activity.

The Ballast Water Convention requires that the concentration of viable organisms in ballast water must be less than 10 per mL. Therefore, analysis of the individual microalgae is necessary. This paper presents a novel method of detecting the cell activity and counting the number of single microalgae cells in a microfluidic chip based on non-modulated chlorophyll fluorescence. The chlorophyll fluorescence detection system consists of a microfluidic chip as sample platform, a laser diode as the light source and a photodiode as the photo-detector. The effects of key parameters on the chlorophyll fluorescence intensity of the microalgae samples were investigated. The activity of the microalgae is determined by the signal peak of the chlorophyll fluorescence. Comparison experiments of five different living and dead algae species (*Chlorella vulgaris*, *N. closterium*, *Platymonas subcordiformis*, *P. delicatula* and *Dunaliella salina*) were performed using the developed fluorescence detection system. The detection limit for the minimum size of microalgae using the developed system is also explored in this paper.

## 2. Experimental Section

### 2.1. Fluorescence Detection System

**Figure 1.**
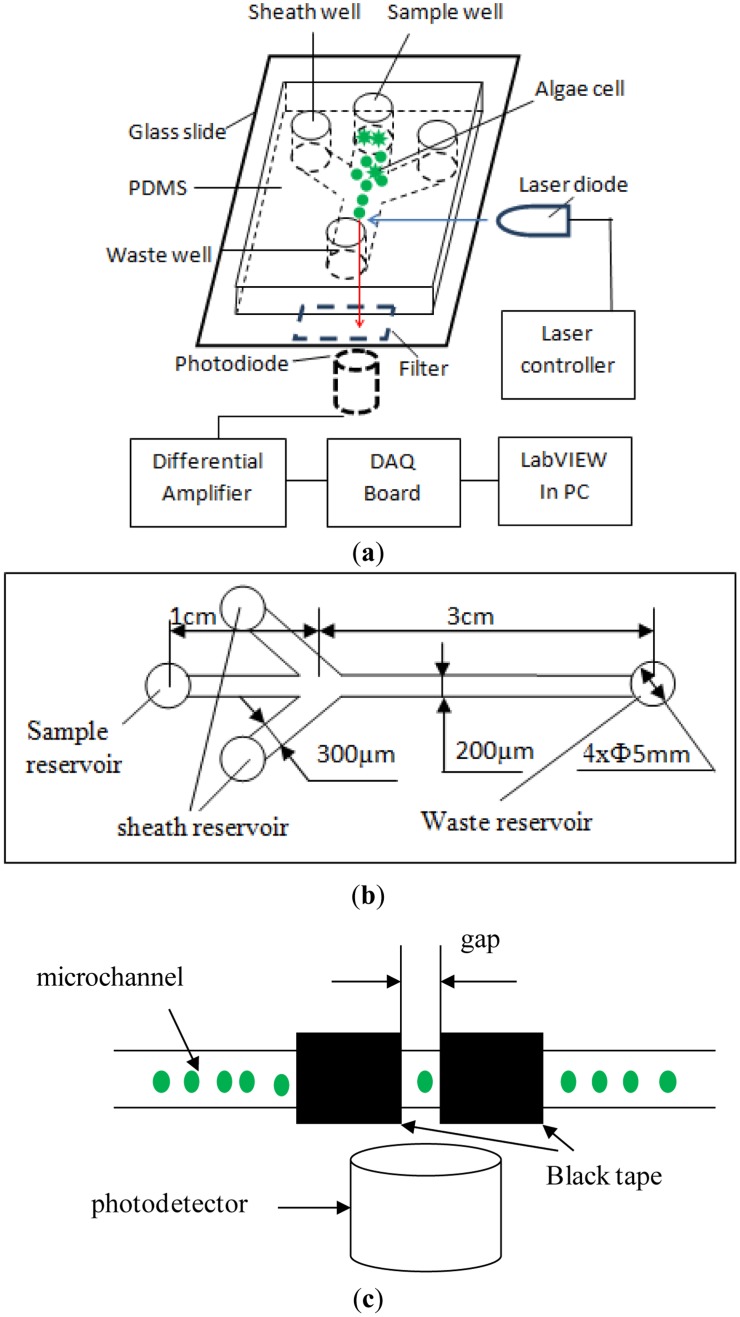
(**a**) The schematic diagram of the chlorophyll fluorescence detection system; (**b**) the dimensions of the microfluidic chip; and (**c**) the schematic diagram of the detection spot.

The chlorophyll fluorescence of single microalgae cells in a microfluidic chip was detected in our lab by using a self-designed optical detection system, as illustrated in Figure 1. The detection system consists of an excitation light source, a microfluidic chip platform, a photo-detector and a data acquisition and processing unit. According to the excitation and the emission spectrum of chlorophyll fluorescence of microalgae, a laser diode (LD) (DL-488-050, wavelength of 488 nm, Shanghai Xilong Optoelectronics Technology Co., Ltd., Shanghai, China) is used as the excitation light source. A photodiode (S8745-01, Hamamatsu, Bridgewater, NJ, USA) is chosen to detect the chlorophyll fluorescence. The output voltage of the photodiode corresponds to the chlorophyll fluorescence intensity. As illustrated in Figure 1c, the emission light is limited to a small detection spot of 200 μm width that is formed by two pieces of black tapes on a thin glass slide placed on the top of the photodetector. This is to minimize the possibility of detecting multiple cells at the same time. A differential amplifier circuit was designed to improve signal-to-noise ratio of the output signal from the photodiode. An emission filter (ET680, Chroma, Bellows Falls, VT, USA) is mounted between the photo-detector and the sample. For acquiring and processing the signals from the amplifier circuit, a NI USB-6259 DAQ board (National Instrument, Austin, TX, USA), is used and a LabVIEW program (National Instrument, Austin, TX, USA) is coded.

### 2.2. Microfluidic Chip Design and Fabrication

The illustration of the designed microfluidic chip is shown in Figure 1. This microfluidic chip consists of a fork-shaped microchannel and four reservoirs. The dimensions of the microfluidic chip are shown in Figure 1b. The width and the length of the microchannel from the sample reservoir to the junction are 200 μm and 1 cm, respectively. The two branch microchannels are 300 μm wide and 1 cm long and connected to two liquid reservoirs for hydrodynamic flow focusing. The two laminar flow streams from the two sides will force the sample cells to move in a single line, and pass through the optical detection spot one by one along the microchannel center line. The straight microchannel from the junction to the waste reservoir is 200 μm wide and 3 cm long. The detection spot is at the middle of the straight channel. All the microchannels have a height of 40 μm and all reservoirs have a diameter of 5 mm and a depth of 2 mm.

The microfluidic chip was made of a Polydimethylsiloxane (PDMS) plate and a glass slide (24 mm × 50 mm × 0.15 mm, Citotest Labware Manufacturing Co., Ltd., Haimen, China) following the standard soft-lithography protocol [36]. A layer of SU-8 photoresist was coated on a bare silicon wafer by a spin coater (G3P-8, Cookson Electronics Equipment, Indianapolis, IN, USA). Then a photomask containing the designed microchannel structure was put on the silicon wafer and irradiated by ultraviolet light. The SU8 master was obtained after post-baking and developing processes. A mixture of PDMS and curing agent was mixed, degased and poured on the master, and then heated at 75 °C for 5 h in the vacuum oven under normal pressure (Isotemp model 280A, Fisher Scientific, Pittsburgh, PA, USA). Finally, the PDMS replica was peeled off from the master. Holes were punched on the PDMS layer for wells. The PDMS layer with the microchannel structure was bonded onto a glass slide after being treated by oxygen plasma for 50 s in a plasma cleaner (PDC-30G, Harrick Plasma, Ithaca, NY, USA). A position marker was used to facilitate the alignment of the detection spot in the microchannel and the photodetector.

### 2.3. Sample Preparation

#### 2.3.1. Culture of Microalgae

Six algae species (*Karenia mikimotoi* Hansen, *Chlorella vulgaris*, *N. closterium*, *Platymonas subcordiformis*, *P. delicatula* and *Dunaliella salina*) were obtained from the Liaoning Sea Fisheries Research Institute (LSFRI). Each algal species was cultured alone in a conical flask of enriched seawater medium [37], which was shaken once every three hours. They grew in a CO_2_ incubator [MGC-300A, Yiheng Technical Co., Ltd., Dalian Maritime University (DMU), Dalian, China] under a photoperiod of 12 h. The temperature was maintained at 20 °C and the illumination intensity was 3,000 lx.

#### 2.3.2. Microalgae Killed by Heat

First, 1 mL microalgae solution was put into a 1.5 mL centrifuge tube and the centrifuge tube was placed in a beaker of 50 mL in volume. The beaker was filled with water (30 mL) and then put on a heater. The heater was set at 120 °C. The temperature of the algae solution increased gradually until the temperature reached 100 °C. The sample solution is kept at 100 °C for 15 min. After being treated by heat, the microalgae solution was cooled down to room temperature and examined under a microscope to see if all the microalgae cells do not move. In order to ensure all the microalgae cells were dead, the microalgae solution was inspected once every 2 h for movement. The number of the microalgae cells was also examined once every 24 h. After 48 h, if no movement was observed and the number of the microalgae cells was not changed, it was concluded that all the microalgae cells were dead.

## 3. Results and Discussion

### 3.1. Chlorophyll Fluorescence signals of Individual Living and Dead Microalgae Cells

**Figure 2.**
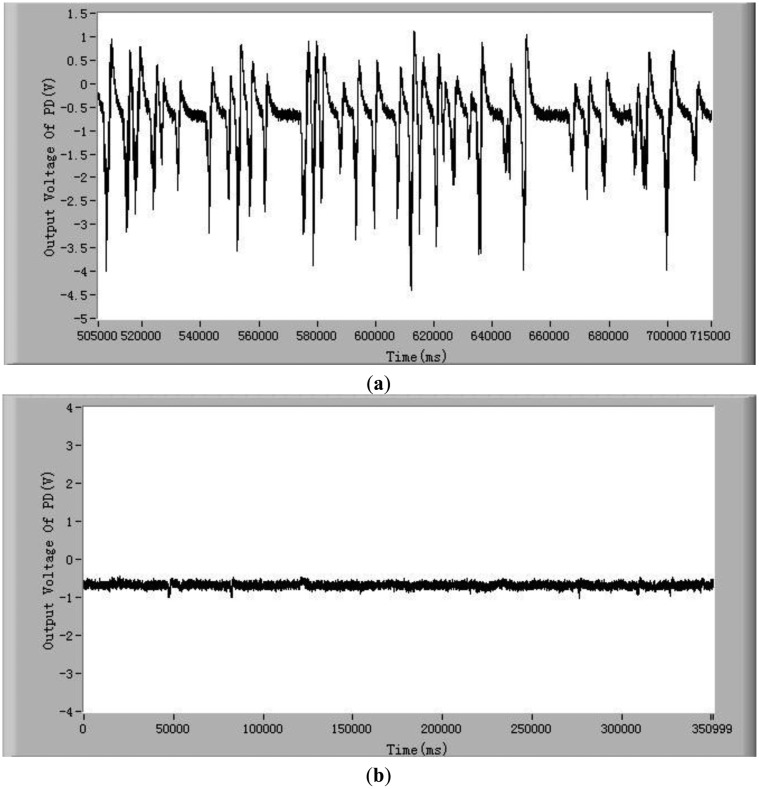

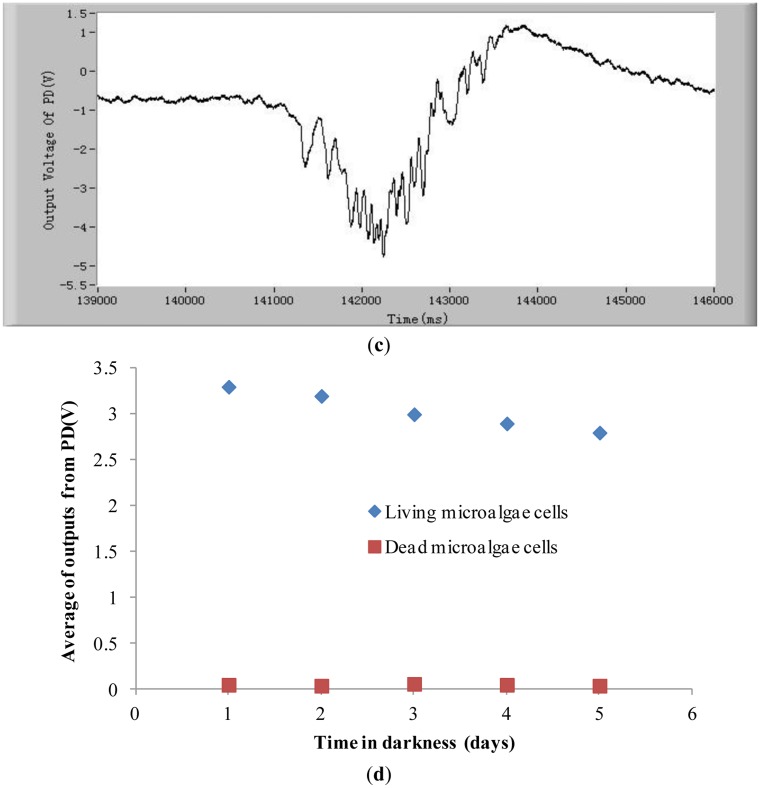
Typical chlorophyll fluorescence signals of *Karenia mikimotoi* Hansen (**a**) individual living cells (**b**) dead cells (**c**) an enlarged view of a living cell signal. And (**d**) average chlorophyll fluorescence intensity of living and dead cells after being treated in darkness. The excitation light power is 2 mW and temperature is 21 °C.

In order to demonstrate that the method and the system developed in this study can detect the chlorophyll fluorescence of an individual cell, both alive and dead *Karenia mikimotoi* Hansen cells were tested. In these tests, the excitation light power is 2 mW and temperature is 21 °C. Typical chlorophyll fluorescence signals of individual living microalgae cells are shown in Figure 2a. The chlorophyll fluorescence intensity represents the activity of a microalgal cell. The higher the chlorophyll fluorescence intensity, the higher is the viability of the cell. As can be seen from this figure, each pulse in the curve represents a living microalgae cell. When a microalgae cell is dead, the photosynthesis is stopped and the chlorophyll fluorescence intensity is theoretically zero [38]. In practice the chlorophyll fluorescence intensity of a dead microalgae cell is close to the background noise level, which is shown in Figure 2b. Comparing Figure 2a,b, one can see the great difference in chlorophyll fluorescence intensity between the alive and dead microalgae cells. Furthermore, for a live cell, there are many small peaks in a big pulse, as shown in Figure 2c.

These small peaks are due to the different relaxation processes in the photosynthetic organs. The mechanism of these relaxation processes is very complex [39,40]. The amplitudes, quantities and intervals of these small peaks of species of microalgae cell are different. Therefore, characterizing these small peaks may be a potential method for classifying microalgae. In order to judge if the detection system developed in this study is able to differentiate between stressed but still living microalgae cells and dead microalgae cells, experiments of chlorophyll fluorescence intensity of living and dead cells after being treated in darkness were conducted. The results are shown in Figure 2d. The results show that there still exist obvious differences between living microalgal cells and dead microalgal cells after being treated in darkness. The average chlorophyll fluorescence intensity of living microalgae cells decreases with the increase of time of being in darkness owing to the fact the activity of cells degrades, however this average fluorescence intensity is still greater than that of dead cells.

In this study, three measures were taken to minimize the detection errors caused by overlapping cells. First, hydrodynamic flow focusing was employed to make cells pass by the detection spot one by one in a single line, as shown in Figures 1a,b. Secondly, as illustrated in Figure 1c, a small gap on top of the photo-detector was used to greatly reduce the size of the detection spot, and hence the possibility of detecting multiple cells at the same time. Finally, the number of cells in the detection region at the same time generally is proportional to the concentration of cells. In order to reduce the chance of multiple cells co-existing at the detection spot, the concentrations of all algae samples tested in this study were kept low, 1 × 10^4^ cells/mL. Using *Karenia mikimotoi* Hansen as a sample, an experimental investigation was conducted to examine the correlation of the concentration of cells with the presence of multiple cells in the detection region of the microfluidic chip and the detection system used in this study. It should be realized that the amplitude of the chlorophyll fluorescence signal generated by two or more cells will be twice or more than that of chlorophyll fluorescence signal produced by only one cell. Therefore, by observing the chlorophyll fluorescence signals, we can judge whether cell overlapping occurs. The results are shown in Table 1 and indicate that the overlap may not occur when the concentration of microalgae cells is less than 5 × 10^4^ cells/mL.

### 3.2. Comparison of Chlorophyll Fluorescence of the Different Kinds of Living and Dead Microalgae

**Figure 3.**
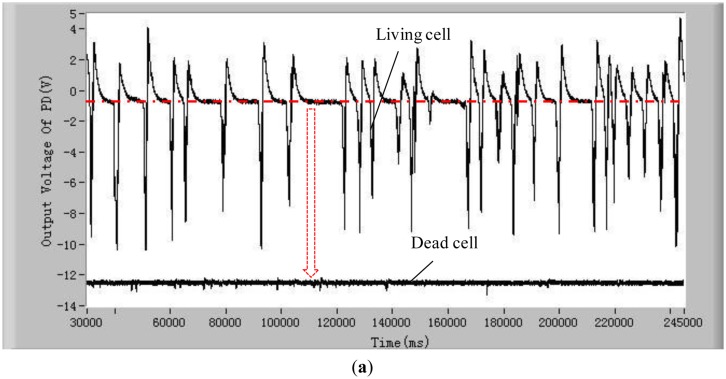

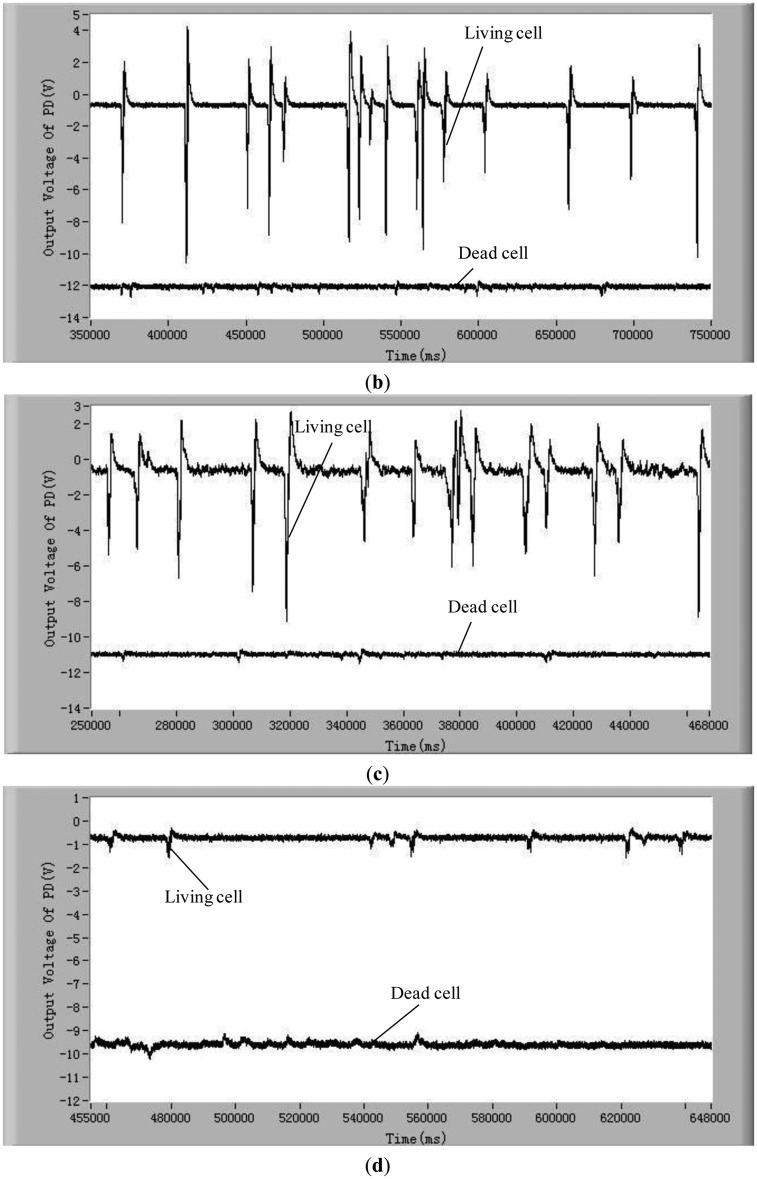

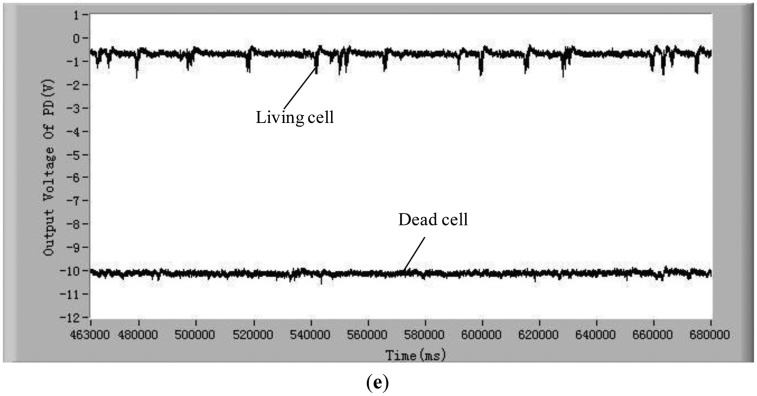
Measured chlorophyll fluorescence of the living and dead microalgae cells of five microalgae species: (**a**) *Platymonas subcordiformis;* (**b**) *Dunaliella salina;* (**c**) *P. delicatula*; (**d**) *N.closterium*; and (**e**) *Chlorella vulgaris*. The excitation light power is 8 mW and the temperature is 21 °C.

To validate the developed method and system for distinguishing the difference between living and dead cells, experiments with five different kinds of living and dead microalgae cells (*Dunaliella salina; Platymonas subcordiformis; P. delicatula; N. closterium; Chlorella vulgaris*) were conducted. The results are shown in Figure 3. All these tests were done under the same conditions (the concentration of microalgae cells is 1 × 10^4^ cells/mL, the excitation light power is 8 mW and the temperature is 21 °C). The shape and sizes of these algal cells are shown in Table 2.

In order to show clearly the difference of chlorophyll fluorescence between the living and dead microalgal cells in one figure, the position of the voltage signal of the chlorophyll fluorescence of the dead microalgae cells was moved vertically downwards to a lower position. For clarity, this position shift is illustrated only in Figure 3a. The results in these figures clearly show that there are many pulses from the living microalgae cells and there is no any pulse from the dead microalgae cells. This is because when a living microalgae cell passes through the optic detection point, the cell is excited by the laser, chlorophyll fluorescence was emitted from the cell and detected by the photodiode. The detected signal was converted to an electronic pulse through a differential amplifier circuit, therefore, each pulse represents a living cell and the number of the pulses is equal to the number of the living cells. In this way, the living cells can be counted by examining the number of measured electronic pulses.

### 3.3. Limit of Detection

**Figure 4.**
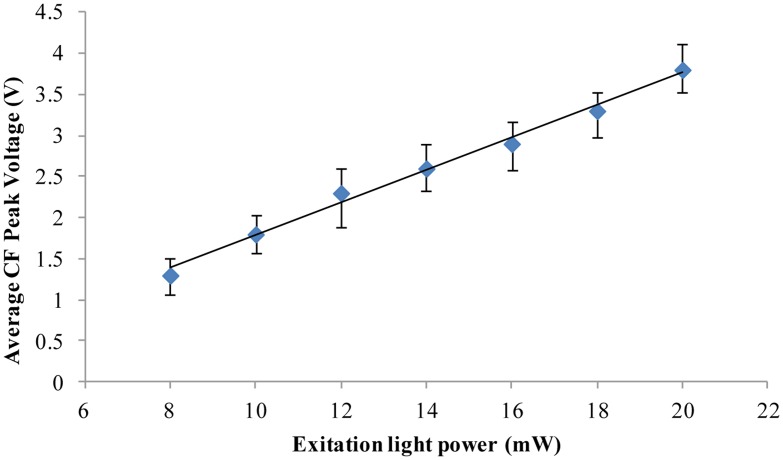
The relation between average chlorophyll fluorescence intensity of the microalgae cells (*Chlorella vulgaris)* and the power of the excitation light. Data are the averages [mean ± Standard Error (S.E.)] of twenty-one repeated measurements. Temperature is 21 °C.

*Chlorella vulgaris* is the smallest commonly available microalga. From the above-described experiments, the results show that for *Chlorella vulgaris* of 3 μm diameter living and dead microalgae cells can still be distinguished. It should be noted that the chlorophyll fluorescence intensity will be affected by the excitation light power. Taking *Chlorella vulgaris* as an example, the relationship between the chlorophyll fluorescence intensity and the excitation light power was investigated by the designed fluorescence detection system. The results are shown in Figure 4. The results show that the chlorophyll fluorescence intensity will increase with the increase of the excitation light power. 8 mW excitation light power was used in the above-reported experimental results (Figures 2 and 3). When the excitation light power is increased, the chlorophyll fluorescence intensity output will also be improved. Therefore, we believe that even smaller microalgae cells can be detected to distinguish the alive cells and the dead cells by increasing the excitation light power and improving the signal analysis method.

### 3.4. Dependence of Cell Activity on Temperature

**Figure 5.**
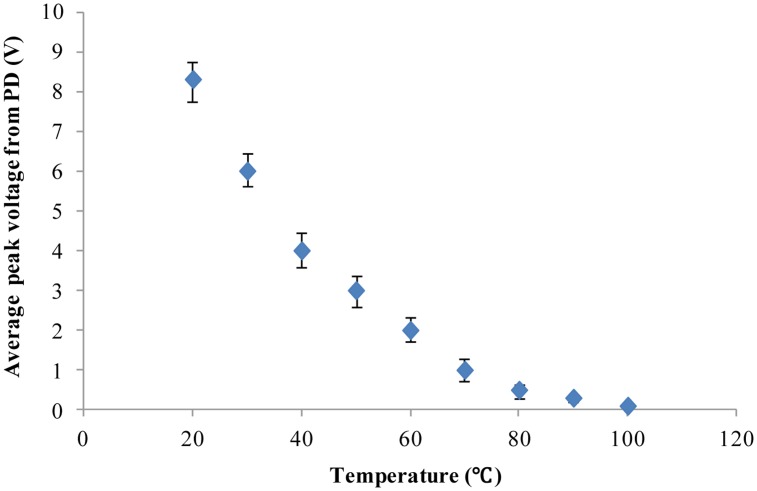
The response of chlorophyll fluorescence intensity of the microalgae cells (*platymonas subcordiformis*) to temperature. The excitation light power is 8 mW. Data are the average (mean ± S.E.) of twenty-one repeated experiments.

As demonstrated above, our developed method and system based on chlorophyll fluorescence can distinguish between individual alive microalgal cells and dead microalgal cells. Furthermore, our cell chlorophyll fluorescence sensor chip was applied to study the dependence of microalgal cell activity on temperature. The microalgal cells (*Platymonas subcordiformis*) were placed in an environment with a constant temperature for 15 min before being tested for chlorophyll fluorescence on the chip. Nine different temperatures (20, 30, 40, 50, 60, 70, 80, 90 and 100 °C) were used in this study. The chlorophyll fluorescence of the microalgae cells were measured by the fluorescence detection system developed in this study. The results are shown in Figure 5. Clearly, with the increase of the treatment temperature, the chlorophyll fluorescence intensity declines continuously until it is close to the background noise level. This is in agreement with others' findings of the temperature dependence of the photosynthesis activity [41,42]. The results show that our chlorophyll fluorescence LOC sensor can also quantitatively detect the microalgal cell activity.

## 4. Conclusions

In this study, a new microfluidic method for detecting single living microalgal cells based on chlorophyll fluorescence was presented. The correlations between chlorophyll fluorescence intensity and the life status and the activity of the cells were investigated. The results show that the developed system based on chlorophyll fluorescence can not only detect the living status of single microalgal cells, but also can evaluate quantitatively their viability. It was demonstrated that microalgal cells of 3 μm in diameter can be detected. The novel features of the biosensor described here include: (1) the biosensor can detect the viability of microalgal cells in ballast water on a microfluidic chip by using the chlorophyll fluorescence of the cells; no labeling is required. (2) Compared with the existing methods such as flow cytometry, the developed biosensor has the advantages of low cost, small size, and simple operation. This biosensor has great potential for *in situ* examination of ballast water.

## Figures and Tables

**Table 1.** The cell overlap as a function of the concentration of microalgae cells.

**Concentration of cells (cells/mL)**	**Overlap condition**
1 × 10^3^	No overlap
5 × 10^3^	No overlap
1 × 10^4^	No overlap
5 × 10^4^	No overlap
1 × 10^5^	Overlapped
5 × 10^5^	Overlapped
1 × 10^6^	Overlapped

**Table 2.** The shape and size of the tested microalgal cells.

**Species**	**Shape**	**Size**
*Platymonas subcordiformis*	Spheroid	Length: ∼15 μm; Width: ∼10 μm
*Dunaliella salina*	Spheroid	Length:∼12 μm; Width: ∼8 μm
*P. delicatula*	Spheroid	Length: ∼10 μm; Width: ∼6 μm
*N.closterium*	Meniscus	Length:∼11 μm; Width: ∼3 μm
*Chlorella vulgaris*	Spheroid	Diameter: ∼3 μm
